# Recalibration of the stress response system over adult development: Is there a perinatal recalibration period?

**DOI:** 10.1017/S0954579423000998

**Published:** 2023-08-29

**Authors:** Mariann A. Howland

**Affiliations:** Institute of Child Development, University of Minnesota, Minneapolis, MN, USA

**Keywords:** perinatal, pregnancy, postpartum, recalibration, stress

## Abstract

During early life-sensitive periods (i.e., fetal, infancy), the developing stress response system adaptively calibrates to match environmental conditions, whether harsh or supportive. Recent evidence suggests that puberty is another window when the stress system is open to recalibration if environmental conditions have shifted significantly. Whether additional periods of recalibration exist in adulthood remains to be established. The present paper draws parallels between childhood (re)calibration periods and the perinatal period to hypothesize that this phase may be an additional window of stress recalibration in adult life. Specifically, the perinatal period (defined here to include pregnancy, lactation, and early parenthood) is also a developmental switch point characterized by heightened neural plasticity and marked changes in stress system function. After discussing these similarities, lines of empirical evidence needed to substantiate the perinatal stress recalibration hypothesis are proposed, and existing research support is reviewed. Complexities and challenges related to delineating the boundaries of perinatal stress recalibration and empirically testing this hypothesis are discussed, as well as possibilities for future multidisciplinary research. In the theme of this special issue, perinatal stress recalibration may be a mechanism of multilevel, multisystem risk, and resilience, both intra-individually and intergenerationally, with implications for optimizing interventions.

## Introduction

The stress response system is an ancient physiological mechanism, maintained by natural selection and highly conserved across vertebrae and mammalian species (Crespi & Denver, 2005). The stress system coordinates the body’s responses to both threats and opportunities, mobilizing physiological and psychological systems to respond to fluctuating environmental conditions and to maintain homeostasis (McEwen, 2007). Several lines of empirical evidence establish that long-term response profiles of the stress system are programmed or calibrated to match the quality of social and physical environments experienced during early life-sensitive windows of heightened neural plasticity (Del Giudice et al., 2011; Gunnar & Howland, 2022). Such windows include the prenatal, infancy, and pubertal periods. By encoding key features of the environment and translating this information to influence a range of physiological and behavioral responses, the stress response system is a critical mediator of development and adaptation over the lifespan (Crespi & Denver, 2005; Del Giudice et al., 2011). Evidence that this system can be calibrated to harsh pre- and post-natal environments and then *recalibrated* when conditions shift to supportive (or vice versa) highlights its potential as a multilevel, multisystem mechanism of risk and resilience over development (Cicchetti, 2010; Masten et al., 2021).

In this paper, I integrate several lines of theory and research to hypothesize that the perinatal period is an additional sensitive window in the lifespan of birthing individuals, during which the stress response system is calibrated to match the quality of the current environment, whether stressful or supportive. This proposal focuses specifically on the hypothalamic-pituitary adrenal (HPA) axis, a key neuroendocrine arm of the stress response. While the entirety of the stress response is complex and involves extensive coordination and interaction between brain regions, the HPA axis, and both branches of the autonomic nervous system (see Ulrich-Lai & Herman, 2009), the scope of this paper is limited to the HPA axis, given the predominant emphasis on this system in the early life stress, stress recalibration, and perinatal stress literatures. The term “perinatal” is used here to refer to pregnancy, lactation, and early parenting. Not all birthing people identify as women or mothers, yet the research base on which this hypothesis is built has centered on biologically female individuals. While the terms women and mothers are used in this paper, future research should consider both sex and gender identity as they intersect with gestation, childbirth, and early parenting. Further, it is yet to be established which of these stages of the perinatal period are necessary for stress recalibration, so this hypothesis is relevant for non-birthing parents as well.

The paper begins with an overview of the structure and function of the HPA axis. Next, theories of the developing stress response system are reviewed, including concepts of sensitive periods, programming, and adaptive calibration. New findings that puberty may be a sensitive period for recalibration of the stress response after early life calibration are summarized. Then, integrating and extending these conceptual and empirical literatures, the possibility of perinatal recalibration of the stress response system is outlined by discussing the 1) life history significance, 2) heightened neural plasticity, and 3) marked adaptations in HPA axis activity characteristic of the perinatal period. Lines of empirical evidence that will be needed to substantiate the perinatal stress recalibration hypothesis are proposed, and relevant extant findings are summarized. Complexities and challenges related to defining the boundaries of perinatal stress recalibration and empirically testing the hypothesis are then described, followed by possibilities for future empirical investigation. In line with this special issue, the paper concludes by highlighting the relevance of this hypothesis for multilevel, multisystem approaches to understanding and promoting resilience during the perinatal period. Ultimately, resilience-promoting prevention and intervention efforts informed by a perinatal stress recalibration lens can focus on improving lifespan health and wellbeing and disrupting the intergenerational transmission of early life stress and adversity.

## Anatomy and physiology of the HPA axis response

The neuroendocrine stress response system (SRS) comprises two anatomically distinct but functionally integrated circuits: the sympathetic-adrenal-medullary (SAM) axis and the hypothalamic-pituitary-adrenal (HPA) axis (for a detailed review, see Gunnar & Vazquez, 2006). The SAM axis is a central component of the fast-acting sympathetic nervous system, which mounts the body’s short-lived “fight or flight” response to physical and psychological challenges (see Ulrich-Lai & Herman, 2009). The HPA axis has a relatively greater threshold for activation compared to the SAM axis and mounts a slower, longer-lasting response to highly salient environmental threats and opportunities (Sapolsky et al., 2000). Beyond situations that threaten bodily harm, the axis appears to be particularly sensitive to social-evaluative and uncontrollable threats (Gunnar et al., 2009; Miller et al., 2007). Given these features, it is not surprising that the HPA axis has been the primary target of research aimed at understanding how the SRS can “transduce environmental signals into developmental responses” (Crespi & Denver, 2005, p. 46) to influence lifespan health and behavior.

**Figure 1.**
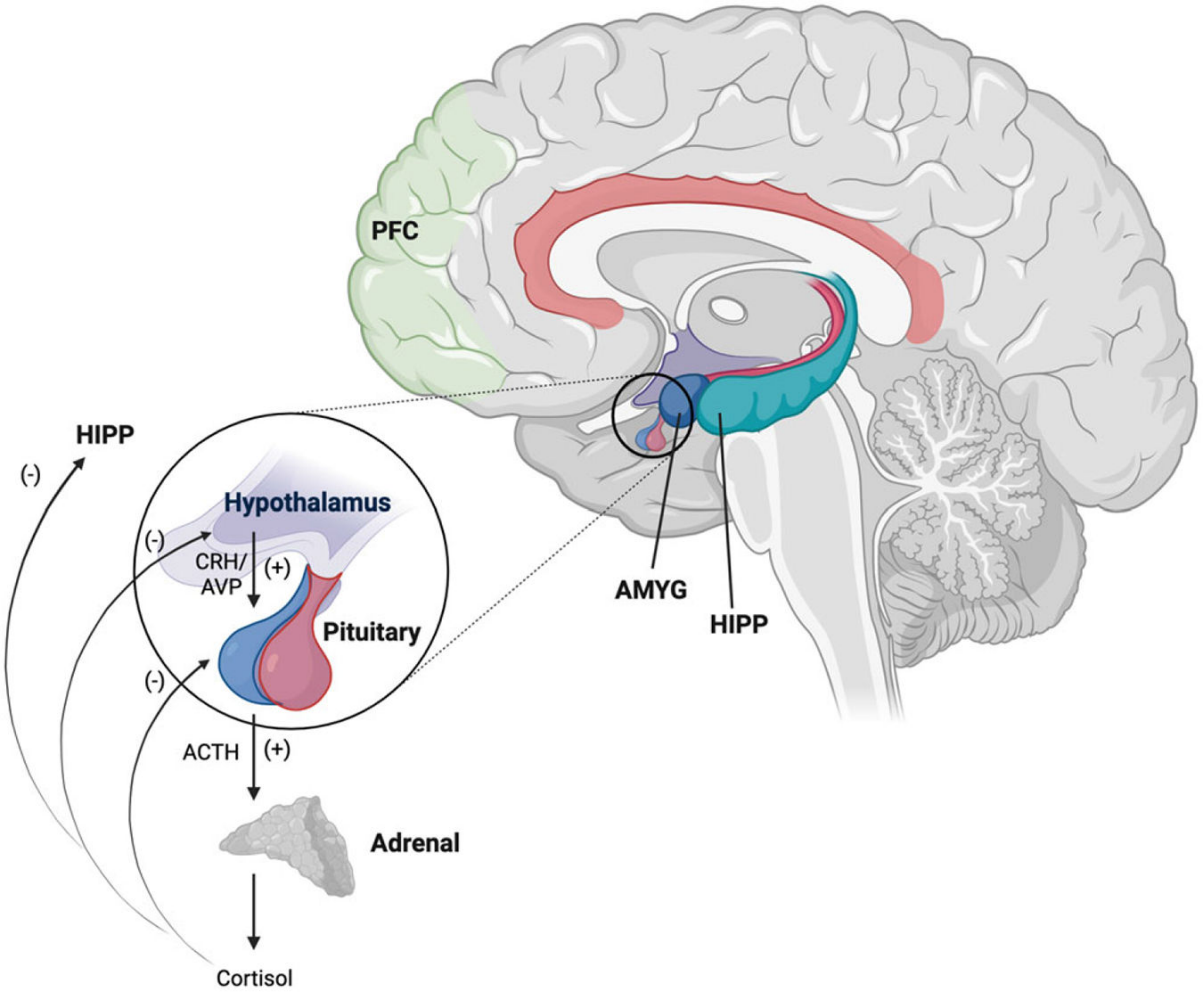
Schematic representation of the hypothalamic–pituitary–adrenal (HPA) axis. In response to challenge, corticotropin-releasing hormone (CRH) is synthesized in the paraventricular nucleus (PVN) of the hypothalamus and, along with arginine vasopressin (AVP), is secreted into the hypophyseal portal blood. CRH binds to its receptors on pituitary corticotropes, stimulating production of adrenocorticotrophic hormone (ACTH). ACTH then enters the bloodstream and induces secretion of cortisol from the adrenal cortex, which mobilizes the brain and body systems to respond to the challenge. Under normal conditions, elevated circulating cortisol inhibits further HPA axis activity (−) by binding to its receptors at the level of the hypothalamus, pituitary, and hippocampus. CRH-producing neurons in the PVN of the hypothalamus are innervated by afferent projections from multiple brain regions, including the amygdala, hippocampus, medial prefrontal cortex, and brainstem, which provide excitatory (+) and/or inhibitory (−) input. ACTH = Adrenocorticotropic hormone; AMYG = amygdala; AVP = arginine vasopressin; CRH = Corticotropin-releasing hormone; HIPP = hippocampus; PFC = prefrontal cortex. Created with BioRender.com.

The primary regulator of the HPA axis is the neuropeptide corticotropin-releasing hormone (CRH; Smith & Vale, 2006; Aguilera & Liu, 2012; see Figure 1). CRH-producing neurons in paraventricular nucleus (PVN) of the hypothalamus are innervated by afferent projections from multiple brain regions, including the amygdala, hippocampus, medial prefrontal cortex, and brainstem (see Herman et al., 2020). When inhibitory inputs are lifted and/or excitatory inputs are increased, CRH, along with arginine vasopressin (AVP), is secreted into the hypophyseal portal blood. CRH binds to its receptors on corticotropes of the anterior pituitary, stimulating production of adrenocorticotrophic hormone (ACTH) and other bioactive peptides. AVP participates by potentiating the effects of CRH on ACTH release. ACTH then enters the bloodstream and induces secretion of the glucocorticoid steroid hormone cortisol from the zona fasciculata of the adrenal cortex. Cortisol levels in plasma and saliva peak approximately 10–30 minutes after the onset of the challenge, but its impacts on the brain and body continue for longer periods (Sapolsky et al., 2000; Ulrich-Lai & Herman, 2009).

In plasma, cortisol binds with high affinity to cortisol-binding globulin (CBG), leaving approximately 5–10% of cortisol unbound (free) to act on target tissues (Cizza & Rother, 2012). Circulating free cortisol exerts rapid, non-genomic effects to affect a variety of tissues within the cardiovascular, endocrine, immune, and nervous systems (see Gray et al., 2017). Cortisol’s slower, gene-mediated effects occur through its binding to two types of receptors: the type I, high-affinity mineralocorticoid receptor (MR), and the type II, low-affinity glucocorticoid receptor (GR), which are widely distributed throughout the brain and body. Cortisol has a 10-fold higher affinity for MRs than for GRs, so at basal concentrations of cortisol, MRs are largely occupied, and GRs remain largely unoccupied (de Kloet et al., 1998). MRs are proposed to regulate the tonic actions of cortisol, including its normative circadian (e.g., increase in cortisol upon awakening, decline over the day) and pulsatile rhythms, as well as set points of HPA axis activation. GRs are increasingly occupied during periods of elevated cortisol in response to challenge and regulate negative feedback inhibition of the axis following its activation (Smith & Vale, 2006; de Kloet et al., 1998). While the basal and stress-reactive components of the HPA axis are distinguished, they are fundamentally related, as basal levels of cortisol prepare or prime the system to respond to challenges (Sapolsky et al., 2000).

In binding to its receptors, cortisol directly regulates gene expression in a range of tissues and organs, with effects of elevated circulating cortisol including activation and regulation of cardiovascular, metabolic, and immune systems; inhibition of feeding, reproductive, and growth functions; and enhancement of attention, arousal, learning, and memory processes (see O’Connor et al., 2000; Sapolsky et al., 2000). The genomic effects of cortisol on target tissues can take hours to establish and may persist for extended periods (Sapolsky et al., 2000). Cortisol has many actions in the brain and shapes neural development and plasticity across the lifespan, especially in GR and CRH receptor-rich brain regions regulating the SRS (e.g., hypothalamus, hippocampus, amygdala, prefrontal cortex; see Dedovic et al., 2009; Herman et al., 2020; McEwen, 2012). High levels of circulating cortisol inhibit further HPA activity by binding to receptors at the level of the hypothalamus, pituitary, and hippocampus (Herman et al., 2020; Smith & Vale, 2006; de Kloet et al., 1998), and, normally, this terminates the stress response. In binding to its receptors in these brain regions, cortisol acts to encode and store information about the frequency, type, and severity of challenges and opportunities in the environment (Del Giudice et al., 2011), shaping the sensitivity of this negative feedback loop.

While HPA axis activation is adaptive in the face of acute threat or opportunity, prolonged or chronic elevations in cortisol render GRs persistently occupied. Excessive GR occupation and associated alterations to the structure and function of brain regions regulating the HPA axis can impair inhibitory and/or increase excitatory neural input to the axis, resulting in a pattern of hyper-responsivity (see Herman, 2013). Severe, chronic stress resulting in frequent and prolonged SRS activation is proposed to impose “wear and tear” on the brain and body systems, increasing vulnerability for disease (McEwen, 2012). Chronic SRS activation may eventually lead to a desensitization to stressors over time to protect the brain and body from the deleterious effects of prolonged cortisol elevations, reflected in a progressive blunting or pattern of hypo-responsivity (see Miller et al., 2007). If cortisol levels are chronically low, too few MRs are occupied to prepare the SRS and other systems to respond effectively to challenges (Sapolsky et al., 2000; de Kloet, 1991). However, when occurring outside of a sensitive period of heightened neural plasticity (see next section), such adjustments to stress system activity tend to occur gradually and may remit or reverse once the stressor is removed (Gabard-Durnam & McLaughlin, 2019; Koss & Gunnar, 2018).

Challenges that occur during sensitive periods may have prolonged impacts on the system’s activity, even when conditions change later in development. Altered activity HPA axis is considered a primary mechanism by which early life stress (ELS; e.g., deprivation, maltreatment) “gets under the skin” and impacts long-term health (Berens et al., 2017; Koss & Gunnar, 2018; McEwen, 2012; Miller et al., 2011). Both hyper- and hypo-responsive profiles are observed among individuals exposed to early life and/or chronic stress, with evidence that these patterns can persist into adulthood (see meta-analyses by Brindle et al., 2022; Hakamata et al., 2022; Perrone et al., 2023). These patterns are in turn associated with a host of physiological and behavioral outcomes (see Berens et al., 2017; Koss & Gunnar, 2018).

## Theories of the developing stress response system

How do and why do experiences during specific early developmental windows exert particularly strong and long-lasting effects on the stress response, and why might the perinatal period be another such window in the lifespan of biologically female individuals? Several concepts and theories central to understanding this process are reviewed here (see also Gunnar & Howland, 2022).

### Sensitive periods

During sensitive periods of heightened neural plasticity in development, neural circuits undergo rapid change and are more responsive to experience (Knudsen, 2004; Luby et al., 2020). Plasticity is an intrinsic property of the brain that facilitates its adaptation to changing environments over the lifespan (Cicchetti, 2010; Gluckman et al., 2019). While sensitive periods are often conceptualized as windows of vulnerability to environmental insults, they are increasingly recognized to also constitute resilience-promoting windows of opportunity. Conditions experienced during a sensitive window of development impart effects on brain and body systems which can persist long after the window has closed and even when conditions change (Takesian & Hensch, 2013; Gabard-Durnam & McLaughin, 2019). If an experience (or lack thereof) occurs during a sensitive period, its effects are modifiable later in development, just not as readily, perhaps until another sensitive period. Sensitive periods are distinguished from critical periods in that during the latter, an absence or presence of inputs results in permanent change (Knudsen, 2004). The effects of environments experienced during sensitive windows of development are likely to depend on the brain regions exhibiting plasticity at that time and to be brain region-specific (Chattarji et al., 2015; Gee & Casey, 2015; Luby et al., 2020).

The fetal and infancy periods, collectively termed the “first 1,000 days” (conception through 2 years of age), are identified as sensitive windows for the SRS. In these early life phases, HPA axis activation set points and response profiles are established, and experiences can exert influences on the system that persist into adulthood (see Howland et al., 2017; Koss & Gunnar, 2018). Several theories have been advanced to interpret the meaning of these potentially enduring impacts.

### Developmental origins, fetal programming, and postnatal development

During prenatal life, the basic architecture of the highly plastic and rapidly developing fetal brain is established (Stiles & Jernigan, 2010), and HPA axis set points appear to be shaped by maternal signals of *ex utero* environmental conditions (see Howland et al., 2017). David Barker’s Developmental Origins of Health and Disease hypothesis (DOHaD; see Barker, 2007) is substantiated by several decades of human and animal research showing that developmental trajectories, including that of the HPA axis, are “programmed” by prenatal exposures, with links to later physical and mental health outcomes (O’Donnell & Meaney, 2017). From a DOHaD or programming perspective, alterations in HPA axis function resulting from prenatal stress are usually interpreted to reflect deviations from expected or typical patterns of development, increasing risk for later disease.

Brain development continues well into infancy and toddlerhood and is characterized by high rates of synaptogenesis and myelination (Stiles & Jernigan, 2010). The HPA axis response is still developing during early postnatal life and is fundamentally regulated by the consistency and responsivity of early caregiving relationships (Hostinar et al., 2014; Koss & Gunnar, 2018). In humans, threats to connections with attachment figures (e.g., through parental separation, insecure attachment relationships, family conflict) are associated with patterns of SRS regulation and consequences for physiology and behavior from infancy into adulthood (see Brindle et al., 2022; Hakamata et al., 2022; Perrone et al., 2023). Severe early social deprivation and neglect in the form of institutional rearing is repeatedly linked with HPA axis hypo-responsivity (see Gunnar & Reid, 2019). This blunting appears to persist for years after these children are adopted into supportive homes, providing robust evidence for an early sensitive period for SRS calibration. Pioneering work by Gunnar et al. (2019) has leveraged the time-limited severe stress and marked changes in social and physical environmental conditions experienced by these children to probe for possible SRS recalibration during later sensitive windows (see Pubertal Stress Recalibration section below), a hypothesis challenging to test in humans given that ELS often continues into later life.

### Predictive adaptive responses and adaptive calibration

Evolutionary-developmental theories posit that trajectories of stress system development reflect *adaptive* adjustments which engender physiological and behavioral functioning that is well-suited to environmental conditions, whether harsh or supportive (Del Giudice, 2012; Gluckman et al., 2019). The Predictive Adaptive Response (PAR) hypothesis posits that environmental signals during sensitive windows (e.g., fetal period) provide a prediction or “forecast” of the quality of the environment in which the individual will subsequently develop, with the course of development thus reflecting preparation for the *anticipated* environment (see Bateson et al., 2014). When the prediction is correct, physiology and behavior are advantageous in the ensuing environment. Maladaptive outcomes may occur if the actual environment does not match the PAR. Alternatively, an adaptation may initially promote positive development but eventually result in maladaptation if the quality of the environment changes (Gluckman et al., 2019). This idea of mismatch is highly relevant for potential later-life windows of SRS recalibration.

The evolutionary-developmental Adaptive Calibration Model (ACM; Del Giudice et al., 2011) also is grounded in the perspective that developmental alterations in stress system function are adaptive, including those that differ from “typical” profiles. Central to the ACM is the idea that individuals respond in biologically and behaviorally adaptive ways not just to supportive environments but also to unsupportive ones. The ACM is consistent with multilevel, multisystem definitions of resilience as the “capacity of a dynamic system to adapt successfully through multisystem processes to challenges that threaten the function, survival, or development of the system” (Masten et al., 2021, p. 524).

The ACM proposes that the SRS is calibrated to match environmental conditions, giving rise to inter- and intra-individual adaptive variation in life history (LH) behaviors (Del Giudice et al., 2011; Ellis et al., 2009). LH behaviors refer to competing, energetically expensive life functions, namely growth, bodily maintenance, and reproduction. According to life history theory, based on environmental conditions, tradeoffs are made in the allocation of limited resources (e.g., energy, nutrients, time) to these competing functions over the lifespan, with an individual’s constellation of tradeoffs comprising their LH strategy. The ACM postulates that the SRS is a mechanism of conditional adaptation, influencing LH strategies via gene-environment interplay to encode features of early environments (e.g., harshness, unpredictability, supportiveness) which, over evolutionary time, have reliably predicted the quality of the environment in which individuals will mature (Del Giudice et al., 2011; Ellis et al., 2009). The SRS is assumed to continuously calibrate to match changing environmental conditions across development, feeding back on itself over time to incorporate new information into its long-term pattern of responsivity and becoming more canalized over time (Del Giudice et al., 2011; see also Knudsen, 2004; Walasek et al., 2022). This process of adaptive calibration leads to person-specific, context-dependent stress response profiles and LH behaviors (Del Giudice et al., 2011).

The ACM proposes that LH strategies are particularly likely to undergo substantial change, as mediated by calibration and recalibration of the SRS, during major developmental transitions, termed “developmental switch points” (Del Giudice et al., 2011). Del Giudice et al. (2011) identify key developmental switch points with relevance to SRS calibration and the expression of LH strategies, segmenting development into the prenatal period, infancy, childhood, juvenility (middle childhood), and adolescence. During these transitions, changes in the stress response are expected if environmental conditions have shifted substantially (e.g., from predictable to unpredictable). While the ACM seemingly suggests that the SRS is open to calibration over much of childhood and adolescence, the ELS literature suggests more circumscribed windows of calibration, namely, sensitive periods for the SRS (e.g., fetal period, infancy). Critically, adaptive calibration affords the potential for “repair and reversal” of developmental processes if conditions change from harsh to supportive (Blair & Raver, 2012, p. 313).

## Pubertal stress recalibration

Theories of the developing SRS pertain primarily to the fetal and infancy periods. Growing attention has been directed to the pubertal transition as another major developmental switch point and period of heightened plasticity during which the brain and body may be particularly responsive to environmental threats and opportunities (see Fuhrmann et al., 2015). Puberty involves normative increases in HPA axis reactivity to stressors in both rodents (Romeo, 2018) and humans (see Gunnar & Howland, 2022). Brain regions regulating the SRS also demonstrate significant maturation during the pubertal period, particularly the prefrontal cortex (Delevich et al., 2021). Animal models indicate that the pubertal brain is especially sensitive to the effects of the more prolonged cortisol exposures following stress that occur in this period (see Romeo, 2010). This sensitivity is also likely to facilitate greater benefit from supportive experiences (e.g., Colich et al., 2021).

**Figure 2.**
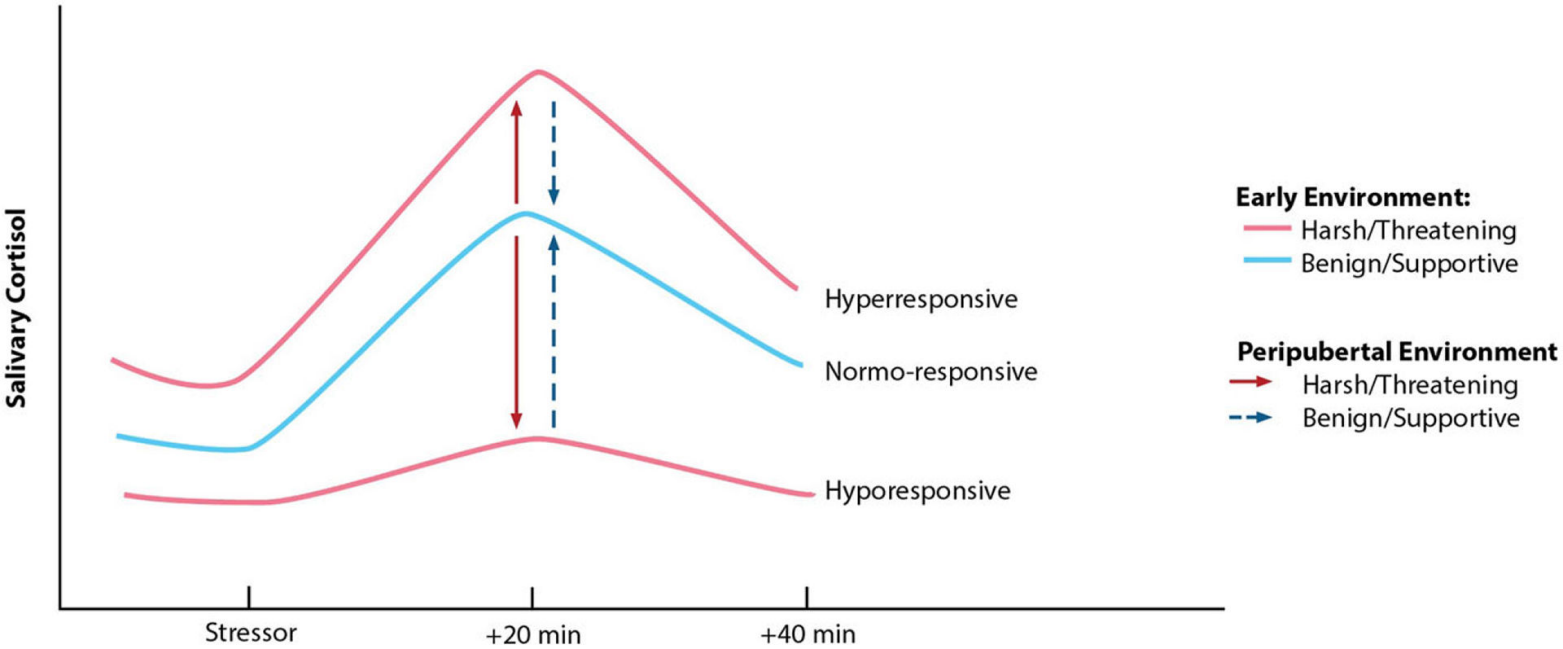
Hypothesized pattern of pubertal recalibration of stress responsivity (Gunnar & Reid, 2019). If the quality of environmental conditions during pubertal development is benign/supportive, children with hyperresponsive or hypo-responsive profiles may shift toward a more normative or typical response pattern. Conversely, if the peripubertal environment is harsh/threatening, an individual’s profile may remain hyper-or hypo-responsive or become further exaggerated in either direction. Reprinted with permission from Gunnar & Reid (2019).

Work by Megan Gunnar offers the first evidence in humans of pubertal recalibration of the stress response, evaluating the hypothesis that if environmental conditions have changed substantively (e.g., from harsh to supportive), the system may recalibrate to function in a way more adaptive or typical in the new context (e.g., become more or less reactive; see Figure 2). Building on her work showing that previously-institutionalized (PI) youth continue to exhibit blunted stress reactivity for years following adoption into more supportive, resourced homes, Gunnar et al. (2019) demonstrate that within-individual advances in pubertal stage are associated with increases in cortisol reactivity to a laboratory psychosocial stressor for these children, reflecting a shift from hypo-responsivity to greater responsivity. By late puberty, PI youth exhibit reactivity profiles comparable to those of non-adopted, comparison youth. Several other groups also report findings suggestive of pubertal recalibration of the HPA axis (King et al., 2017; VanTieghem et al., 2021; Zhang et al., 2021). Importantly, because plasticity likely renders the pubertal HPA axis more responsive to both positive and negative exposures, its activity may become further blunted or exaggerated if conditions continue to be stressful, or if conditions shift from supportive to harsh (see King et al., 2017). Rodent studies offer causal support for pubertal SRS recalibration with changed conditions, for better or for worse (see Romeo, 2018). For example, prenatal stress-induced HPA axis hyper-activity is reversed following environmental enrichment during the peri-adolescent period (Morley-Fletcher et al., 2003). Interestingly, this enrichment has no impact on HPA axis reactivity among rodents that did not experience prenatal stress, suggesting that the system does not need to recalibrate when conditions are unchanged across developmental periods (findings from Gunnar et al., 2019 are aligned with this possibility). Whether periods of stress recalibration exist beyond puberty remains to be explored.

## Support for the possibility of perinatal stress recalibration

In advancing the ACM, Del Giudice et al. (2011) speculate about developmental switch points in the lifespan beyond puberty, suggesting menopause for women and middle age for men. They additionally state that “other factors may also contribute to strategic adjustment during adult life, even without qualifying as identifiable switch points. An event of special significance may be represented by the birth of one’s first child: not only does it signal (some degree of) reproductive success, but it is known to affect hormonal functioning in both sexes and could thus directly interact with the endocrine systems that regulate the expression of LH strategies” (p. 1571).

This section will discuss theory and empirical evidence to substantiate that the perinatal period (here, inclusive of pregnancy, lactation, and early parenting) is a developmental switch point in the lifespan of biologically female individuals, during which the SRS may recalibrate to match current conditions. Animal studies demonstrate that neurobiological effects of exposure to offspring alone (e.g., among pup-exposed virgin rats) appear to be distinct and/or less pronounced as compared to changes conferred collectively by pregnancy, lactation, and early caregiving (Kinsley & Lambert, 2008; Pawluski et al., 2009a; Pawluski & Galea, 2007), so it is plausible that pregnancy is a critical component of the window. As a basis for formulating and testing a perinatal stress recalibration hypothesis, three literatures are reviewed here, with the aim to draw parallels to theory and research on windows of calibration earlier in life: 1) evolutionary-developmental perspectives on the significance of reproduction as a LH behavior and its relevance to calibration, 2) evidence of heightened neural plasticity during this period, and 3) evidence of the marked, adaptive alterations in HPA axis activity characteristic of this phase.

### Perinatal period as a developmental switch point

Evolutionary-developmental theory designates reproduction as a critical lifespan activity involving significant energetic investment. Tradeoffs are made across the female lifespan with respect to prioritizing growth versus reproduction, survival versus reproduction, and current versus future reproduction, which, from an LH perspective, serves to maximize the perpetuation of one’s genes in future generations (Coall & Chisholm, 2010; Ellis et al., 2009; Perlman, 2019). Maternal reproductive strategies (e.g., timing of first pregnancy, number of pregnancies, offspring birthweight) are shown to be adaptively shaped by environmental conditions and psychosocial stressors experienced during early life-sensitive periods (Chisholm et al., 2005; Coall & Chisholm, 2010; Kuzawa, 2007). Thus, early life stress is likely relevant not only to stress system function and potential recalibration during a perinatal period in a woman’s lifespan but also to the timing and number of perinatal periods she will experience.

Each individual pregnancy constitutes a developmental switch point during which maternal biological resources are redirected and social roles are shifted. The substantial allocation of metabolic energy and other physiological resources to support gestation, fetal growth, childbirth, lactation, and the onset and maintenance of caregiving behavior requires tradeoffs that may occur at the expense of other physiological processes (Perlman, 2019), with these physiological demands described as a “stress test” for body systems (see Williams, 2003). Maternal adaptations over pregnancy include dramatic changes across neural, endocrine, cardiovascular, pulmonary, immune, and metabolic systems (see Napso et al., 2018; Torgersen & Curran, 2006). These adaptations represent the mother’s evolutionarily optimal “investment” in her offspring (in evolutionary biology, terms like “investment” are used to denote mechanisms of natural selection and do not imply conscious behavior; Del Giudice, 2012). This investment is proposed to be calibrated by the quality of the current environment (e.g., its safety, predictability, resource availability, social support), the woman’s somatic resources (e.g., age, physical health, nutritional state), the “quality” (e.g., health) and quantity of current offspring, the “vigor” of the fetus, and the likelihood of additional offspring in the future (Coall & Chisholm, 2010; Ellis et al., 2009; Haig, 1996). The maternal SRS likely acts as a central mechanism of this calibration.

### Perinatal period as a sensitive period

The perinatal period is increasingly recognized as a sensitive window of development not only for the fetus but also for the mother, and it is also acknowledged as a period involving not only heightened vulnerability but also enhanced opportunity for the environment to shape brain and behavior (Davis & Narayan, 2020; Glynn et al., 2018; Howland & Cicchetti, 2021; Orchard et al., 2023). Although pregnancy and lactation are transient, rodent and human studies demonstrate that, as in the fetal and pubertal periods, massive fluctuations in sex and stress steroid hormones exert organizing effects on the brain, resulting in lasting changes to brain structure and function, physiology, and behavior (see Glynn et al., 2018; Luders et al., 2022). As in early life sensitive periods, the neural plasticity of the perinatal period may allow the brain to be highly responsive to experience, in which case stressful or supportive conditions could exert stronger and more lasting effects on the SRS during relative to outside of this period.

A rich rodent literature demonstrates that the pregnant and lactating female brain is remodeled by many of the same mechanisms used to sculpt neural circuits during early development. Neuronal, dendritic, and synaptic plasticity occurs in numerous brain regions, most notably in the medial preoptic area of the hypothalamus, but also regions known to directly regulate the HPA axis, particularly the hippocampus, but also the PVN of the hypothalamus, the amygdala, and the prefrontal cortex (for reviews see Pawluski et al., 2022; Slattery & Hillerer, 2016). Corticosterone (the rodent equivalent of cortisol) appears to play an important role in the altered dendritic morphology and neurogenesis observed with reproductive experience (Leuner et al., 2007; Pawluski et al., 2009b, 2015).

Rodent data thus far suggests that the patterning of changes in the hippocampus shifts over the perinatal period, though studies are primarily cross-sectional. Late pregnancy and early lactation are linked with increased spine density in pyramidal neurons in the CA1 region of the hippocampus (Kinsley et al., 2006). Shifts are seen postpartum, with these neurons undergoing significant dendritic pruning at the time of weaning in primiparous rats (Pawluski & Galea, 2006). Hippocampal cell proliferation and immature neuron survival are significantly decreased in primiparous (first birth) and multiparous (previously given birth) rats as compared to nulliparous (no births) rats, beginning in midgestation and extending into at least the early postpartum period (Darnaudéry et al., 2007; Eid et al., 2019; Leuner et al., 2007; Pawluski & Galea, 2007). Such changes potentially reflect greater neural efficiency and/or a tradeoff to allocate metabolic resources toward lactation (Leuner et al., 2007; Medina & Workman, 2020). This does not appear to be due to pup exposure alone, as pup removal 24 hours after birth does not influence new neuron survival 3 weeks later. Further, among nulliparous females, exposure to pups instead results in *increased* rates of cell proliferation and survival (Pawluski & Galea, 2007), and a hormone-simulated pregnancy suppresses cell proliferation (Green & Galea, 2008). Motherhood also involves increased hippocampal long-term potentiation which persists well into aging (Lemaire et al., 2006). In the rodent medial amygdala (Rasia-Filho et al., 2004) and PFC (Leuner & Gould, 2010), dendritic spine density is enhanced among postpartum rats, and in the rat hypothalamus, changes in synaptic plasticity are evident in oxytocinergic neurons in the PVN during delivery and lactation (Marlin et al., 2015; Shams et al., 2012). One longitudinal study using magnetic resonance imaging techniques documents transient early postpartum increases in gray matter concentrations in multiple brain regions, including the PVN, hippocampus, and amygdala, with the magnitude of increase positively associated with the amount of pup-directed care (Barrière et al., 2021).

A growing number of neuroimaging studies in humans document structural and functional alterations in the brain over pregnancy and the postpartum period, including changes in gray matter volume, cortical thickness, and cortical surface area (for review, see Luders et al., 2022). One study demonstrates that first pregnancy results in substantial reductions in gray matter volume in frontal, cingulate, and temporal cortices which are observable until at least 2 years post-pregnancy and comparable to volumetric reductions seen over the pubertal phase (Carmona et al., 2019; Hoekzema et al., 2017, 2022). A small subset of these participants was followed to show that changes persist until at least 6 years post-pregnancy (Marítinez-García et al., 2021). These gray matter reductions are positively associated with postpartum maternal attachment ratings and neural responsivity to pictures of one’s own baby. Consistent with rodent findings (Barrière et al., 2021), *increases* in gray matter volume (Kim et al., 2010; Lisofsky et al., 2019; Luders et al., 2020) and cortical thickness (Kim et al., 2018) are observed over the first few months postpartum in women. Thus, the maternal brain appears to demonstrate both decreases and increases in size which may be temporally patterned (e.g., decreases in pregnancy and increases postpartum) and/or regionally specific. This is broadly consistent with rodent evidence regarding differences in the directionality of hippocampal neuronal changes depending on perinatal stage (i.e., pregnancy vs. postpartum).

Brain remodeling over the perinatal period is critical for the onset and maintenance of maternal caregiving behavior but also has implications for health more broadly (see Medina & Workman, 2020; Puri et al., 2023). Conceptual and empirical cross-species work considers motherhood as a form of environmental enrichment, as interactions with offspring who provide rich sensory and social inputs serve to increase environmental novelty and complexity and necessitate the development of new skills (Orchard et al., 2023; Pawluski et al., 2016). In rodents, reproductive experience results in enhancements in learning and memory and reductions in anxiety behaviors which persist into old age (see Macbeth & Luine, 2010). A parallel human literature suggests that maternal brain changes involve some tradeoffs, enhancing certain abilities (e.g., responsivity to threats and to infant-related stimuli, spatial associative memory; Callaghan et al., 2022; Hoekzema et al., 2022) at the (potentially temporary) expense of other cognitive functions (e.g., verbal memory; see Orchard et al., 2023; Ziomkiewicz et al., 2019). And while the relationship between parity and later-life disease appears to be complex (see Puri et al., 2023), emerging evidence shows that reproductive plasticity has neuroprotective effects, with a history of childbirth related to enhanced cognitive performance and less apparent brain aging in mid- to late-life (Ning et al., 2020; Orchard et al., 2020; de Lange et al., 2020). Collectively, these findings underscore that the perinatal period is a developmental epoch in the lifespan that engenders lasting change to brain and behavioral functioning.

The degree of neural plasticity observed in brain regions regulating the HPA axis in both rodents and humans alludes to the possibility that inhibitory and/or excitatory inputs to the axis may be altered in pregnancy and lactation, with potential persisting effects. This prospect is further supported by the massive normative changes in HPA axis function over gestation and lactation detailed in the next section. One potentially important question is the extent to which environmental influences on perinatal brain plasticity operate by experience-expectant versus experience-dependent neural mechanisms, or both (see Gabard-Durnam & McLaughlin, 2019). This could speak to the type of environmental stimuli which may have heightened impact on the SRS and associated brain regions during the perinatal period (see Fuhrmann et al., 2015). Findings from rodent models clearly indicate that the maternal brain “expects” to experience pup stimuli through lactation and the provision of other caregiving behaviors. An absence of this expected input (e.g., in the case of separation or loss) may to some degree disrupt the brain remodeling and typical changes in HPA axis activity underlying the onset and maintenance of effective caregiving behaviors (see Demarchi et al., 2021), with potentially diminished opportunity for their development once the sensitive window has closed. Broader environmental dimensions (e.g., harshness, unpredictability) may instead operate mostly via experience-dependent mechanisms, though the brain may “privilege” certain features of the environment which signal the capacity for investment in caregiving behaviors (e.g., cues of social support). Determining what information is most salient to the developing maternal brain can inform hypotheses about the potential long-term effects of stressful and supportive experiences during this window on the stress system.

### Perinatal adaptations in HPA axis activity

Substantial changes in the basal activity and responsivity of the stress system, particularly the HPA axis, are evident during the perinatal period. These normative alterations are involved in regulating gestation, fetal maturation, lactation, and caregiving behavior (Almanza-Sepulveda et al., 2020; Liggins, 1994; Pawluski et al., 2009b). During pregnancy, changes are largely mediated by the growth of a new neuroendocrine organ, the placenta. As the interface between mother and fetus, the placenta secretes multiple peptide and steroid hormones into maternal circulation, most of which are identical to those produced by the brain and other endocrine organs in the non-pregnant adult (Napso et al., 2018). These hormones target the maternal brain and neuroendocrine organs (Arévalo & Campbell, 2020; Napso et al., 2018), acting as allocrine factors (Mesiano, 2019). Typical changes in both HPA axis basal activity and reactivity over the perinatal period are described in detail here, as each will be important to consider when searching for mechanisms of possible stress recalibration during this life phase.

### Basal activity

**Figure 3.**
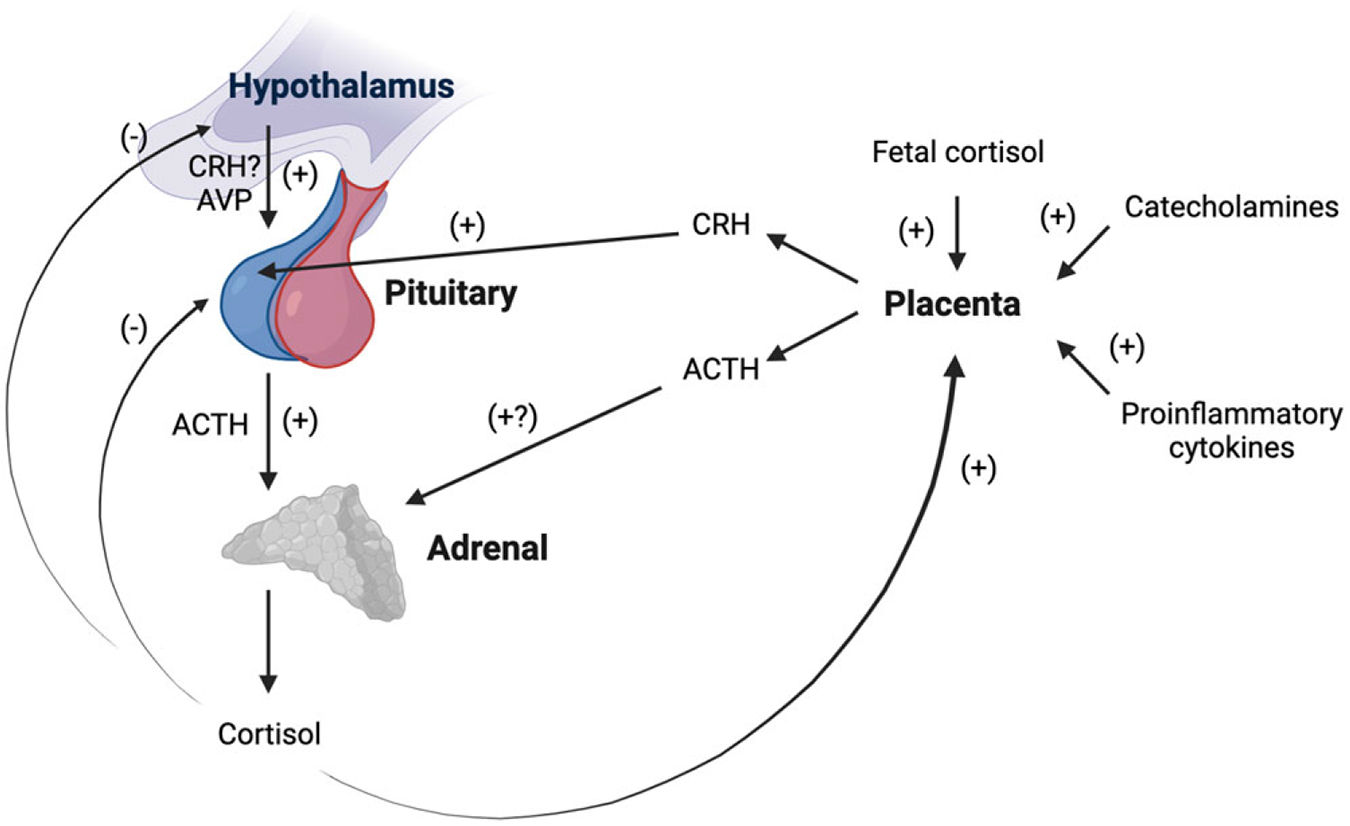
Schematic representation of changes in maternal basal hypothalamic-pituitary adrenal (HPA) axis activity during gestation. The human placenta produces corticotropin releasing hormone (CRH) identical in structure and function to hypothalamic CRH. Placental CRH is released into maternal circulation and rises to up to 1,000 times non-pregnant levels. Increased placental CRH stimulates ACTH release from the pituitary, which doubles in size over gestation. The placenta also appears to produce ACTH which may further increase maternal ACTH levels. The adrenals become progressively hypertrophic as cortisol levels rising 2–5-fold over gestation. The rise in total cortisol is likely due to a combination of increased ACTH, increases in arginine vasopressin (AVP) secretion in the paraventricular nucleus of the hypothalamus, and estrogen stimulation of cortisol binding globulin production. CRH-induced hypercortisolism suppresses hypothalamic CRH production. Maternal cortisol stimulates rather than inhibits placental CRH production (as does fetal cortisol and other major biological stress mediators). Thus, a positive feedback loop is established, resulting in simultaneous rises in placental CRH, ACTH, and cortisol in maternal circulation with advancing gestation. ACTH = Adrenocorticotropic hormone; AVP = arginine vasopressin; CRH = Corticotropin-releasing hormone. Created with BioRender.com.

Numerous mechanisms operate to alter activity at each level of the maternal HPA axis over the perinatal period (summarized in Figure 3; for detailed review, see Howland et al., 2017; Sandman, 2018). CRH, the primary regulator of the axis, is among the hormones produced by the human placenta. Of note, the rodent placenta does not produce CRH, complicating the direct translation of mechanistic rodent studies to human pregnancy (Power & Schulkin, 2006). CRH mRNA is expressed in the human placenta by the seventh week of gestation and is identical to hypothalamic CRH in structure, immunoreactivity, and bioreactivity (see King et al., 2001). Placental CRH is released into both maternal and fetal circulation, with CRH in maternal plasma almost exclusively of placental origin, as circulating hypothalamic CRH is rapidly degraded and largely undetectable (King et al., 2001). Placental CRH production rises exponentially over gestation and increases in maternal plasma up to 1,000 times non-pregnant levels, reaching levels observed in the hypothalamic system during acute psychosocial stress (see Howland et al., 2017; Sandman, 2018). Increased unbound placental CRH in maternal circulation stimulates the synthesis and release of ACTH from the maternal pituitary (Goland et al., 1994; Sandman et al., 2006), which nearly doubles in size over gestation due to lactotroph cell hyperplasia (Gonzalez et al., 1988). Placental CRH also appears to stimulate placental production of ACTH, which may further increase maternal plasma ACTH levels (Petraglia et al., 1987).

Not only do ACTH levels increase, but the adrenal glands are more responsive to ACTH in pregnancy (Nolten & Rueckert, 1981). The adrenals become progressively hypertrophic as cortisol levels rise 2–5-fold over the course of gestation (Jung et al., 2011; King et al., 2022; Murphy et al., 2022; Sandman et al., 2006; Thayer et al., 2018). Due to estrogen stimulation of CBG production (Demey-Ponsart et al., 1982), more cortisol is bound and inactivated, but by the third trimester, free cortisol levels also are elevated (Nolten & Rueckert, 1981; Scott et al., 1990). In addition to placental CRH upregulation of pituitary ACTH secretion, the rise in total and free cortisol levels may be influenced by gestational increases in AVP secretion in the PVN (Magiakou et al., 1996a), which contributes to pituitary ACTH release (Smith & Vale, 2006). Maternal plasma ACTH and cortisol maintain a diurnal rhythm and remain strongly correlated over pregnancy, though the cortisol awakening response is progressively attenuated with advancing gestation (Bublitz & Stroud, 2012; Buss et al., 2009; Entringer et al., 2010; Thayer et al., 2018). Preservation of diurnal rhythmicity is likely due to AVP secretion in the PVN, as placental CRH-induced hypercortisolism suppresses hypothalamic CRH production, and placental CRH does not exhibit a diurnal rhythm (Magiakou et al., 1996a; Schulte et al., 1990). Diminished GR sensitivity may afford maternal organ systems some protection against rising free cortisol levels as gestation advances (Katz et al., 2012).

Rather than inhibiting further CRH expression in the hypothalamus as in the non-pregnant state, maternal cortisol stimulates placental CRH production (as does fetal cortisol and as do other major biological stress mediators such as catecholamines and proinflammatory cytokines; see Howland et al., 2017, Sandman, 2018). Circulating maternal cortisol also does not appear to suppress placental ACTH release as it does in the pituitary (Petraglia et al., 1987). Thus, a positive feedback loop is established, resulting in simultaneous rises in CRH, ACTH, and cortisol in maternal circulation over the course of pregnancy (Goland et al., 1994; Sandman et al., 2006; see Figure 3). Near the very end of pregnancy, CBG and CRH binding protein levels in maternal plasma drop precipitously (Ho et al., 2007; McLean & Smith, 1999). Greater circulating levels of free cortisol and bioactive placental CRH facilitate fetal growth and organ maturation (Liggins, 1994) and relate to the timing of labor and delivery, including risk for preterm birth if excessively elevated (McLean & Smith, 1999; Sandman et al., 2006).

After delivery, maternal cortisol levels gradually decline to non-pregnant levels as the hypertrophic adrenals progressively downsize (Jung et al., 2011; Magiakou et al., 1996b). Immediately after delivery, removal of placental CRH and estradiol inputs to the hypothalamus results in transient adrenal suppression due to low hypothalamic CRH secretion, which is hypothesized to increase vulnerability to psychopathology at this time (Magiakou et al., 1996b). ACTH secretion in response to exogenous CRH is blunted until up to 12 weeks postpartum, while naturalistic basal plasma cortisol levels and cortisol levels following exogenous CRH exposure are in the upper range of normal, likely due to persisting adrenal hypertrophy and elevated CBG concentrations (Magiakou et al., 1996b). At 6–8 weeks postpartum, baseline (pre-stressor) salivary cortisol levels are shown to be lower compared to late pregnancy levels but still elevated relative to non-pregnant controls (Kammerer et al., 2002). Similarly, serial measurement of cumulative cortisol production as indexed in hair reveals that cortisol levels in the first several months postpartum are higher compared to the first trimester but lower relative to the third trimester (King et al., 2022), though it should be noted that hair cortisol likely reflects both basal activity and reactivity of the HPA axis.

Postpartum HPA axis basal activity is further altered in lactation (for review, see Brunton et al., 2008; Hasiec & Misztal, 2018). In humans, suckling is associated with an acute reduction in ATCH and cortisol concentrations, likely in part through oxytocin release which has inhibitory influences on the HPA axis (Altemus et al., 2001; Amico et al., 1994; Handlin et al., 2009). More generally (i.e., not immediately following a feed), at 6–8 weeks postpartum, baseline (pre-stressor) plasma cortisol levels appear to be lower in breastfeeding women compared to both bottle-feeding and non-perinatal women (Kaye et al., 2004). At later postpartum weeks, no differences in baseline cortisol levels are evident among lactating versus non-lactating or postpartum versus non-postpartum women (Altemus et al., 1995, 2001; Tu et al., 2006a). Critically, these studies rely on small sample sizes and pertain to cortisol levels at a single timepoint, limiting the generalizability of findings. The few investigations assessing diurnal cortisol production in lactation, again in mostly small samples, report inconsistent results. Breastfeeding is associated with higher waking cortisol levels (Ahn & Corwin, 2015; Tu et al., 2006b), steeper diurnal slopes (Simon et al., 2016), or no differences in aspects of the diurnal rhythm (Benjamin Neelon et al., 2015; Taylor et al., 2009). A recent study leveraging a large (*N* = 741), cross-sectional sample of nulliparous and postpartum women reports distinct diurnal cortisol patterns based on breastfeeding status (Thayer et al., 2018). Compared to nulliparous women, breastfeeding women between 0–6- and 6–12-months postpartum exhibit lower waking cortisol levels, lower cortisol awakening responses, and lower evening cortisol levels, with some of these differences also observable in women 12+ months postpartum. Conversely, diurnal patterns are mostly similar in nulliparous and non-breastfeeding postpartum women. These findings suggest that lactation-related impacts on basal HPA axis activity may persist well into the postpartum period, though all findings reviewed here pertain to average, between-group comparisons. Whether there are within-individual changes in basal HPA axis activity that extend across and after the postpartum period remains to be established. Alluding to this possibility, Musey et al. (1987) show that serum levels of an adrenal hormone co-synthesized and released with cortisol, dehydroepiandrosterone, are substantially decreased from before a first pregnancy to 7–19 months postpartum, with no such changes observed in nulliparous controls.

### Responsivity

The perinatal period also involves normative changes in responsivity of the HPA axis to challenge. With advancing gestation, HPA axis responsivity is progressively attenuated. By late pregnancy, exogenous CRH administration does not induce an ACTH or cortisol response (Schulte et al., 1990), and physical and psychological laboratory stressors either produce no response (Entringer et al., 2010; Gitau et al., 2001; Hartikainen-Sorri et al., 1991; Kammerer et al., 2002) or a diminished response compared to the non-pregnant state (Fiterman & Raz, 2019). Women’s appraisals of situations as stressful also decline as pregnancy progresses (see Glynn et al., 2008). In seeming contrast, women in late pregnancy show enhanced vigilance to threatening stimuli, reflected in greater PFC activation (Pearson et al., 2009; Roos et al., 2011). It is possible that some of the more downstream HPA axis changes operating over pregnancy (e.g., increases in placental CRH, ACTH, cortisol) prevent such brain activity from exerting appreciable influence on cortisol reactivity to stress. Downregulation of the stress response is proposed to protect the maternal-fetal dyad from damaging effects of excessive cortisol production in the face of significant stress (Glynn & Sandman, 2011; Slattery & Neumann, 2008). Individuals who do not show this dampening appear to be at risk for preterm delivery (Buss et al., 2009; Glynn et al., 2008). The degree to which maternal stress responsivity is attenuated (or not) with advancing gestation may reflect calibration of the maternal (and fetal) stress response to environmental harshness, in which case a lack of downregulation may be adaptive (to an extent, as extreme adaptations in response to severe stress are likely to increase vulnerability to disease; Del Giudice, 2012).

Attenuation of the HPA axis response persists in lactation, with rodent studies providing causal evidence of the stress-buffering effects of suckling (see Brunton et al., 2008; Slattery & Neumann, 2008). In human mothers, ACTH responsivity to exogenous CRH continues to be blunted in lactation (Magiakou et al., 1996b). Cortisol responsivity to laboratory stressors is attenuated among women who have recently breastfed (Altemus et al., 1995; Cox et al., 2015; Heinrichs et al., 2001; though see Altemus et al., 2001), an effect which may be enhanced with increasing parity (see Tu et al., 2006a). One study shows that women at 8 weeks postpartum mount ACTH and cortisol responses to a laboratory stressor initiated at least 100 minutes after they last breastfed, though, without a non-perinatal control group, it is unclear if these responses are still relatively attenuated (Meinlschmidt et al., 2010). This study also finds that more elevated salivary cortisol awakening responses in late pregnancy are associated with more blunted ACTH and cortisol responses to the stressor, suggesting that the link between late pregnancy cortisol production and hypothalamic CRH suppression may extend into the early postpartum period. As with the existing literature related to basal HPA axis activity, no known work has longitudinally assessed within-person profiles of HPA axis responsivity spanning pre- and post-perinatal period, which will be needed to begin to understand whether recalibration occurs during this life phase.

Rodent research points to brain mechanisms of reduced HPA axis responsivity in the perinatal period (see Brunton et al., 2008; Slattery & Neumann, 2008), though, as mentioned, the translation of these findings to humans is limited by the lack of CRH production by the rodent placenta. Rises in cortisol (and placental CRH in humans) in late pregnancy likely enhance HPA axis negative feedback inhibition via suppression of CRH production in the PVN (Johnstone et al., 2000; Magiakou et al., 1996a). CRH mRNA expression in the PVN and CRH binding in the anterior pituitary also are reduced during pregnancy and lactation, contributing to reduced CRH production and release from the PVN (Brunton et al., 2008; Johnstone et al., 2000; Toufexis et al., 1999). Elevations in these hormones may also exert inhibitory influences through increased cortisol binding in the hippocampus, where GR receptor density is elevated in late pregnancy in rodents (Johnstone et al., 2000; Pawluski et al., 2015). Higher circulating placental progesterone is converted to allopregnanolone in the brain, which appears to suppress HPA axis responsivity via upregulation of inhibitory endogenous opioid mechanisms (see Brunton et al., 2008). Excitatory drive to the HPA axis also is downregulated in pregnancy and lactation, reflected in reduced noradrenergic excitatory tone in the PVN of the hypothalamus, including by way of increased production of oxytocin and prolactin, as well as the actions of endogenous opioids (see Douglas et al., 2005; Slattery & Neumann, 2008).

Presumably, this normative HPA axis downregulation is time-limited, as appears to be the case for altered basal activity, though no known studies have directly addressed this (see next section). Also unclear are the implications that this dampened stress responsivity may have for recalibration of the HPA axis during the perinatal period.

## Evidence needed to support the perinatal stress recalibration hypothesis

Three bodies of evidence discussed above, the perinatal period as a 1) developmental switch point and 2) sensitive period which involves 3) dramatic alterations in HPA axis activity, provide a foundation for the possibility of perinatal recalibration of the SRS. Specifically, they draw parallels between this phase of the lifespan and earlier periods of stress system calibration. However, they do not speak directly to the likelihood of perinatal recalibration.

**Figure 4.**
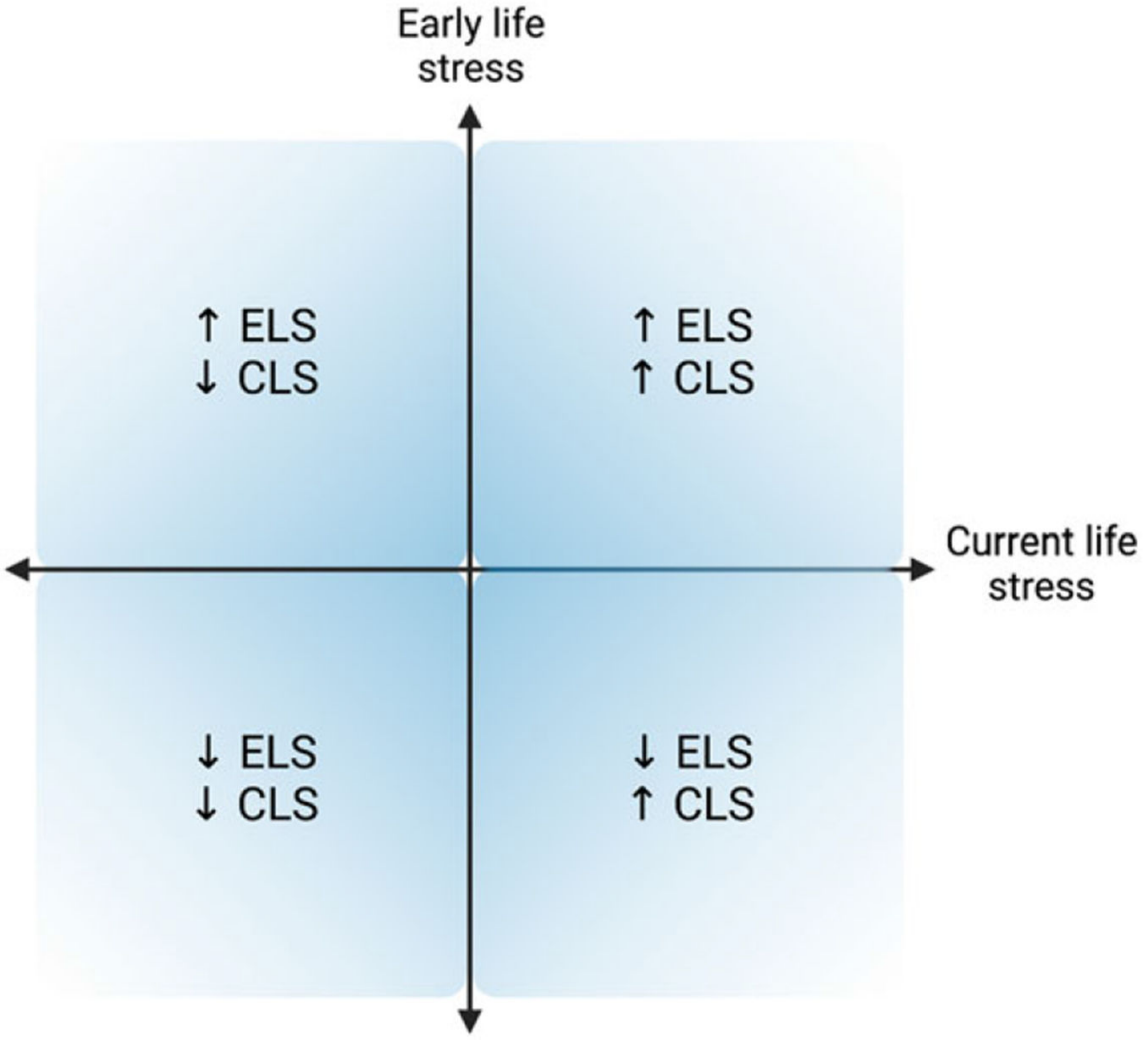
Hypothesized quadrants of early life stress (ELS) and current (perinatal) life stress (CLS) dimensions. Upper right and lower left quadrants represent a “match” between the quality of the early life and current environments, while the other two quadrants represent a “mismatch” between environments experienced during early life and in the perinatal period. Created with BioRender.com.

Testing the perinatal stress recalibration hypothesis requires characterization of both early and current (perinatal) environmental conditions, as well as determination of whether conditions have significantly changed (see Figure 4 for a visualization). This hypothesis posits that if environmental conditions have changed significantly since an earlier period of (re)calibration (i.e., infancy, puberty), the stress response may recalibrate to match conditions experienced during the perinatal period, and these changes would persist and/or be more difficult to modify once the sensitive window has “closed.” As a hypothetical example, a pregnant individual with a history of ELS (e.g., living under conditions of harshness, threat, and/or unpredictability) who has transitioned into a significantly more stable, supportive environment may experience a shift in stress system responsivity from a hyper-active or hypo-active profile to a more normative profile (see Figure 2). If established, perinatal stress recalibration would suggest that the perinatal period is a resilience-promoting window of opportunity for “repair” of stress system activity following ELS, with potential impacts at multiple levels of function (e.g., brain, body, behavior). On the other hand, perinatal recalibration also would enhance vulnerability to the potential consequences of perinatal environmental conditions that as harsh or harsher than previously. The ACM conceptualizes recalibration in either direction as adaptive but acknowledges that calibration to extremely harsh or depriving conditions may result in maladaptation (e.g., risk for disease over the lifespan), as evident in the extensive ELS literature.

What data is needed to substantiate the perinatal stress recalibration hypothesis? Here, I propose several lines of empirical evidence which could speak to the potential for perinatal recalibration, the last of which would provide the most direct and compelling support. Following each piece of supporting evidence, in italics, are examples of opposing evidence that would alternatively suggest the perinatal period is not a window of recalibration (here, early life refers to birth to 18 years of age, as is typically captured with measures of ELS in perinatal research studies):

1. Supporting evidence: Between-individuals, current environmental conditions are associated with differences in perinatal stress responsivity. Early life conditions are either also associated or not associated with perinatal stress responsivity. Specifically, individuals with a history of ELS experiencing supportive conditions during the perinatal period show profiles of stress responsivity more similar or equivalent to those individuals who have experienced continuously supportive environments (i.e., Figure 2), relative to individuals who have experienced continuously adverse environments, and vice versa. Opposing evidence: Current environmental conditions are not associated with differences in the stress response during the perinatal period. If, in this case, early life environmental conditions are associated with perinatal stress responsivity, this would suggest enduring early life influences that are not significantly amenable to adjustment during the perinatal window.
2. Supporting evidence: Between-individuals, the pattern noted in evidence line #1 is present for perinatal individuals but not for never-pregnant/parenting individuals. Opposing evidence: this pattern is either not present for either perinatal or non-perinatal individuals, present for both groups, or present only for non-perinatal individuals.
3. Supporting evidence: Within-individuals, perinatal environmental conditions are associated with alterations in the stress response that persist after the perinatal period. Opposing evidence: Perinatal environmental conditions are associated with alterations in the stress response that are transient and remit after some point.
4. Supporting evidence: Within-individuals, environmental conditions experienced during the perinatal period are more strongly and persistently associated with stress responsivity as compared to conditions experienced in the non-perinatal adult state (e.g., pre-pregnancy). Opposing evidence: Environmental conditions experienced during the perinatal period are equally or less strongly associated with stress responsivity relative to conditions experienced in the non-perinatal state.
5. Supporting evidence: Within-individuals who are followed beginning pre-pregnancy, those who have experienced significant shifts in environmental conditions from earlier periods of (re)calibration show changes in stress responsivity from the pre- to post-perinatal period which are more aligned with current conditions, and these changes persist for at least several years after the perinatal period. Opposing evidence: Those who have experienced significant shifts in environmental conditions do not show persisting changes in stress responsivity from pre- to post-perinatal period. Or, whether or not conditions have shifted, all individuals show persisting changes in stress responsivity from pre- to post-perinatal period.

The following section reviews existing studies in support of evidence of lines 1 and 2. No studies were identified in support of lines 3–5, highlighting critical directions for future research. Possibilities for further empirical investigation are considered below in the Testing the Perinatal Stress Recalibration Hypothesis section.

## Existing evidence in support of perinatal stress recalibration

### Early life and perinatal environmental conditions are associated with differences in the stress response during the perinatal period

ELS and current life stress (CLS) are shown to relate to differences in perinatal HPA axis activity, suggesting that the system may be responsive to both early and current environmental conditions. Several caveats are important to note regarding the following review of this literature. First, while maternal stress system activity is proposed as a key mechanism relating gestational stress with offspring outcomes, surprisingly few studies have directly assessed links between perinatal environments/exposures and maternal stress system function. On the other hand, the last decade has seen a surge of interest in the impacts of maternal ELS on gestational biology. Both bodies of work pertain primarily to basal activity of the HPA axis in pregnancy and lactation, with very few studies measuring responsivity, so studies assessing either or both are included here. Lifetime measures of stress/trauma exposures are not considered in this review (e.g., Dobernecker et al., 2023), as differentiation between ELS and CLS is needed to test questions related to calibration and recalibration. Some investigations measure aspects of both ELS and CLS but do not explicitly report whether both main effects and their interaction were tested in a multivariate model to predict HPA axis function. This limits interpretation of results in terms of their support for perinatal stress recalibration. The current review emphasizes stressful environmental conditions, as studies relating supportive contexts during early life and pregnancy to HPA axis activity are rare (as exceptions, see Bublitz et al., 2014; Thomas, Letourneau, et al., 2018). Most of the investigations described here pertain to between-individual differences, again with a few exceptions (Galbally et al., 2019; King et al., 2022). Many also rely on small sample sizes. These points highlight important limitations to address in future work.

This research faces many of the same challenges evident in the broader ELS-SRS literature, including heterogeneity in measurement of HPA axis activity (e.g., cortisol reactivity to a laboratory stressor, diurnal cortisol patterns, cumulative cortisol production over months); in operationalization of environmental conditions/exposures, both within and across studies; and, as will be made clear below, in the directionality of findings (e.g., stress related to hyper-activity or hypo-activity). Attempting to account for these inconsistencies becomes even more challenging as ELS experiences become more distal in adulthood (see Weems & Carrion, 2007). ELS is often characterized using retrospective, self-report measures, which face validity concerns related to memory- and psychopathology-related bias (see Baldwin et al., 2019). ELS measures used with pregnant samples typically capture experiences spanning birth through adolescence, so they may reflect calibration to both early life and pubertal developmental contexts. Similarly, most studies of CLS during the perinatal period examine levels of *perceived* stress or psychological distress, yet many report weak or null associations between these factors and maternal HPA axis activity (see Khoury et al., 2023; Seth et al., 2016). The broader stress literature suggests that measures of current, more objectively-defined stressors (e.g., major life events), as well as measures of ELS, may interact with stress appraisals/psychological distress to influence patterns of SRS responsivity, including during pregnancy (e.g., Epstein et al., 2020). This work is beyond the scope of this review but will be important to consider in testing the pregnancy recalibration hypothesis. For the purposes of this initial extension of the ACM model to the perinatal period, studies examined here focus on more objective stressors, which nevertheless are limited by their reliance on self-reports. Thirty-eight studies linking ELS and/or CLS with HPA axis functioning during the perinatal period were identified.

In terms of investigations assessing ELS only, ELS is characterized by retrospective reports of exposure to various forms of childhood adversity (e.g., abuse, neglect, family dysfunction, traumatic events, family socioeconomic status) and is associated with both higher (Bublitz & Stroud, 2012; Buss et al., 2016a; Gillespie et al., 2017; Gonzalez et al., 2009; Jairaj et al., 2020) and lower (Carbone et al., 2023; Penner et al., 2023; Shea et al., 2007) basal activity the axis. Three studies that examine HPA axis *responsivity* to laboratory stressors demonstrate that women endorsing early adverse experiences are more likely to be cortisol “non-responders” during pregnancy (Hantsoo et al., 2019) and to show blunted cortisol reactivity at 3–6 months postpartum (England-Mason et al., 2017; Juul et al., 2016; Morrison et al., 2017). Findings pertaining to hair cortisol are complex to interpret, because, as already noted, hair cortisol may capture both basal activity and reactivity of the axis. In hair, childhood trauma is associated with elevated (Buss et al., 2016b; Nyström-Hansen et al., 2019; Schreier et al., 2015; Steudte-Schmiedgen et al., 2023) as well as diminished (Bowers et al., 2018; Penner et al., 2023) cortisol concentrations (though see Orta et al., 2019 for null results). Broeks et al. (2023) report a positive curvilinear (inverted U) relationship between childhood trauma and 0–3-month postpartum hair cortisol levels in a sample selected for antidepressant use in pregnancy. Specifically, relative to women with no history of childhood trauma, women reporting mild to moderate trauma have higher hair cortisol concentrations, while women endorsing severe childhood trauma have comparable or slightly lower concentrations. The authors interpret this pattern to be consistent with the ACM. Overall, this growing body of research suggests that ELS is associated with between-person differences in perinatal HPA axis activity, whether elevated or diminished. More work assessing perinatal stress responsivity is clearly needed, though interestingly, all four known existing studies report that ELS is associated with blunted reactivity. At the broadest level, the literature to date provides initial support for the idea that stress responsivity continues to some extent to reflect calibration to early life conditions.

In studies measuring CLS only, major/traumatic life events (Murphy et al., 2022; Obel et al., 2005) and material deprivation (Thayer & Kuzawa, 2014) are associated with basal HPA axis hyper-activity. Suglia et al. (2010) show that higher scores on composite of several stressors (interpersonal and community violence, discrimination, major life events) relate to hypo-activity of the axis. Several other studies report null findings (King & Laplante, 2015; Kramer et al., 2009; Petraglia et al., 2001). Regarding hair cortisol, King et al. (2022) show that CLS is associated with within- (but not between-) individual differences in cortisol production over the perinatal period. Specifically, within an individual, when CLS is relatively higher, hair cortisol levels also are relatively higher. The remaining CLS and hair cortisol literature consists of null findings (Galbally et al., 2019: Howells et al., 2023; Kramer et al., 2009). CLS-related differences in gestational HPA axis activity are often suggested to reflect deviation from normative pregnancy-related changes, but, in light of the ACM, they may rather reflect an adaptive calibration of the SRS to current environmental conditions.

### Early life and perinatal environmental conditions interact to predict stress responsivity during the perinatal period

Studies that probe whether ELS and CLS have additive or interactive effects on gestational HPA axis functioning are, at this point, the most informative for beginning to understand if pregnancy is a period of stress system recalibration (Bosquet Enlow et al., 2019; Brand et al., 2010; Bublitz et al., 2014; Epstein et al., 2020; Kelsall et al., 2023; King et al., 2008; Stephens et al., 2021; Swales et al., 2018; Thomas, Letourneau, et al., 2018; Thomas, Magel, et al., 2018). Synthesis of existing evidence is difficult given significant methodological variation across studies, as well as complexity in interpreting results both within and across studies, so several examples are described here. A few main effects models show that ELS, rather than or independent of CLS, is associated with elevated HPA axis output (Gillespie et al., 2017; Kelsall et al., 2023). Thomas, Magel, et al. (2018) find no interactions between ELS and CLS, instead showing the ELS but not CLS relates to aspects of diurnal cortisol production. These results are suggestive of enduring ELS effects that are not attenuated by CLS (though see King et al., 2008 for the opposite pattern). Bublitz et al. (2014) document a pattern of increasing (rather than normatively decreasing) cortisol awakening responses over pregnancy among women with more severe child sexual abuse histories that is even more pronounced in the face of perceived difficulties in current family functioning. Conversely, this result also can be interpreted to suggest that more positive family functioning in pregnancy may buffer women with abuse histories from exhibiting this pattern of increase (see Thomas, Letourneau, et al., 2018, for similar results with perceived social support in pregnancy). Two studies considering the interplay between ELS and stress in adulthood (e.g., before the current pregnancy) show corresponding findings: women endorsing more traumatic events in childhood have higher pregnancy hair cortisol levels only if they also report greater numbers of traumatic events in adulthood (Swales et al., 2018), and women with higher ELS show higher waking salivary cortisol levels and blunted cortisol awakening responses only at higher levels of CLS (Epstein et al., 2020). Again, these results conversely suggest that low stress in adulthood is protective for women with a history of ELS, while activity of the axis is further exaggerated when both ELS and stress in adulthood and/or pregnancy are high.

Taken together, findings to date broadly suggest that both ELS and CLS are associated with perinatal SRS profiles, potentially interdependently, beginning to speak to the possibility that perinatal stress responsivity reflects both early life and gestational calibration (though a considerable proportion of studies report null results). Many moderating factors may contribute to inconsistencies in results across investigations (e.g., sample size, participant characteristics), perhaps especially the substantive measurement-related differences within and across studies (e.g., different operationalizations of ELS and CLS). Further, existing findings pertain to cross-sectional (retrospective report of ELS collected at the same time as measures of CLS), between-individual differences and not longitudinal, within-individual change. It remains unclear if environmental conditions during the perinatal period, whether harsh or supportive, have stronger and longer-lasting effects on the SRS as compared to exposures outside of this period. Further, no known findings demonstrate that women whose environmental conditions have shifted significantly from earlier periods of calibration show intra-individual changes in stress responsivity from the pre- to post-perinatal period that persist for several years. In other words, far more evidence is needed to infer that the perinatal period is a window of stress recalibration.

## Testing the perinatal stress recalibration hypothesis

### Complexities and challenges

Before discussing potential strategies for testing the perinatal stress recalibration hypothesis, specifically to establish evidence lines 3–5 outlined above, several factors which add layers of complexity and require consideration are delineated here.

First, important outstanding questions relate to the degree to which environmental conditions (as well as environmental cue reliability; see Walasek et al., 2022) must change, and when in development change must occur, for the SRS to demonstrate recalibration in a later life-sensitive window. A related issue concerns the likelihood of changed environmental conditions from childhood into adulthood. A primary challenge in teasing apart the effects of early and current environmental conditions is that they tend to be correlated across development (see Gunnar & Howland, 2022). Though research suggests that ELS is associated with adult outcomes independent of, or rather than, cumulative stress exposures (see Miller et al., 2011), environmental conditions likely need to change to a certain degree for recalibration of the stress response to occur (see Gunnar, 2020). Even if conditions significantly change, early life experiences may exert lasting impacts on functioning, for example by conferring risk for psychopathology, which, in turn, may shape stress responsivity during the perinatal period (see Koss & Gunnar, 2018; Miller et al., 2007). Alternatively, existing data suggest that early/current life conditions and symptoms of psychopathology may interact to shape stress perinatal stress system activity (Brand et al., 2010; England-Mason et al., 2017; Epstein et al., 2020), though these results do not speak directly to recalibration. Further, multifinality (see Cicchetti, 2010) and between-person differences in neural plasticity (Walasek et al., 2022) over development would suggest that there are individual differences in the extent of recalibration that would occur from one individual to another, even if environmental conditions shift markedly in the same pattern. Risk and resilience factors across multiple levels of analysis are likely to dynamically interact (Cicchetti, 2010; Masten et al., 2021) in shaping the potential for perinatal stress recalibration, both within and between individuals.

In establishing the perinatal period as a window for stress recalibration, it will be important to understand what phases are required for recalibration (pregnancy, lactation, and/or early parenting). For example, rodent research suggests that the cognitive benefits of parity are only evident among rodents with *both* pregnancy and caregiving experience (e.g., not in nulliparous rats given experience with pups or in primiparous rats with no pup experience; Pawluski & Galea, 2007). Other major unresolved questions relate to when the perinatal window would “open” and “close,” as well as when during (or after) the window effects on stress responsivity would become apparent. To elucidate the boundaries and mechanisms of perinatal recalibration, and to understand for whom and to what degree individuals with different perinatal experiences may recalibrate, human studies can include non-birthing parents (i.e., partners, adoptive parents), as well as individuals who are pregnant but do not go on to parent (i.e., in the case of pregnancy loss, surrogacy, or separation). Emerging research documents neuroendocrine changes and enhanced neural plasticity in the paternal brain (see Glasper et al., 2019). Fathers exhibit perinatal reductions in cortical volume and thickness in the default mode network (Kim et al., 2014; Marítinez-García et al., 2023), as well as early postpartum increases in gray matter volume in regions with inputs to the HPA axis (Kim et al., 2014). The later-life benefits of parity on neurocognitive function and brain aging also are observed in fathers (Ning et al., 2020; Orchard et al., 2020). However, there appear to be mixed results regarding whether parenthood influences stress system function in fathers (see Glasper et al., 2019; Storey & Ziegler, 2016). Relevant research on non-biological caregivers is very limited at present, but pregnancy and childbirth are clearly not required for the development of competent caregiving, and similar neuroendocrine mechanisms may be involved (see Bick et al., 2013; Glasper et al., 2019). Overall, these findings in non-birthing parents begin to point to the likelihood of some level of stress recalibration for non-birthing parents, which would likely occur by experience-driven mechanisms (and may thus depend on the extent of participation in caregiving activities; see Abraham et al, 2014). Finally, while perinatal separation/loss has clear impacts on maternal physiology and behavior more generally (see Demarchi et al., 2021), more research is needed to understand its effects on perinatal SRS function and related neural plasticity mechanisms. Several rodent studies demonstrate that separation of a mother from her litter shortly after birth alters at least some of the SRS-relevant neurobiological changes occurring over the perinatal period (see Demarchi et al., 2021), suggesting possible constraints on SRS recalibration in this case.

Additionally, it is unclear if the opportunity for recalibration of the stress response is conferred only by first birth, or if subsequent perinatal periods also constitute windows of recalibration. As stress responsivity is proposed to be canalized over time (Del Giudice et al., 2011), there may be diminishing returns with multiparity, and/or different mechanisms of recalibration with later pregnancies. The maternal brain literature has considered whether the neural plasticity and brain reorganization which “primes” the brain for motherhood is specific to first pregnancy. In rodents, the neural, hormonal, and behavioral changes of pregnancy and lactation vary as a function of parity, with some effects appearing to be cumulative with each additional litter (see Maupin et al., 2016). One human study suggests that the later-life neurocognitive benefits conferred by reproductive experience may not increase monotonically with each additional birth but rather level off at some number (see Ning et al., 2020). In terms of neural mechanisms, primiparous and multiparous rats show different patterns of dendritic remodeling (Pawluski & Galea, 2006) and cell survival (suppressed cell survival is only seen in primiparous rats; Pawluski & Galea, 2007), which the investigators interpret to suggest that the multiparous maternal brain is primed by previous experience to support more efficient and effective responding to infant cues (Pawluski & Galea, 2007). Directly relevant to the stress system, CBG levels are lower and free corticosterone levels are higher among primiparous compared to multiparous rats during lactation, and corticosterone is associated with specific maternal behaviors only among primiparous rats, pointing to a role for elevated cortisol in the first onset maternal behavior (Pawluski et al., 2009a; see also Almanza-Sepulveda et al., 2020). In human mothers, primiparous mothers show a small but significant cortisol response to a laboratory stressor (at least 45 minutes after a feed) that does not differ as a function of feeding type, whereas multiparous breastfeeding women show reduced cortisol responsivity relative to bottle-feeding multiparous women (Tu et al., 2006a). The investigators of this study suggest that the neural mechanisms which modulate HPA axis function in lactation may be heightened with subsequent reproductive experiences, further reducing responsivity of the axis, though replication of these results with larger samples is needed. Whether possible differences in cortisol levels by parity reflect diminished or enhanced potential for stress recalibration with multiparity will be important to investigate.

The perinatal recalibration hypothesis arguably holds particular significance for individuals who experience dramatic shifts in environmental conditions following early life. Adaptive adjustments toward current, more supportive conditions and away from earlier, harsher conditions could render the system more open to the benefits the current environment affords. From the lens of the PAR hypothesis (Bateson et al., 2014), developmental plasticity and stress recalibration during the perinatal period may be a resilience-promoting mechanism to “correct” for mismatch (if conditions change from harsh to supportive, or vice versa), adaptively aligning physiology and behavior to current environment conditions. However, it will be necessary to consider the impacts of recalibration from a multilevel, multisystem perspective, namely that possible tradeoffs at other levels (e.g., physiological, behavioral) may occur with stress recalibration. Such “correction” is likely to require a degree of compensation in the neuroplastic perinatal brain. Typical maternal brain changes appear to involve tradeoffs at the level of cognition (see Orchard et al., 2023; Ziomkiewicz et al., 2019). Cross-lagged positive associations between pubertal stress recalibration and internalizing symptoms among ELS-exposed youth (Perry et al., 2020) suggest that psychological recalibration may lag behind physiological, or that recalibration does not confer benefits across all levels of function and may involve some cost or increased vulnerability (DePasquale et al., 2021). This finding suggests that maternal mental health symptoms should be carefully charted to determine if perinatal stress recalibration is longitudinally predictive of maternal psychopathology; if such a link were established, it would suggest that perinatal stress recalibration is one mechanism that increases women’s risk for psychopathology during the perinatal period.

An additional (and major) layer of complexity is that the perinatal period is a dyadic process, with the maternal prenatal SRS serving as a signal to the calibrating fetal stress system regarding the quality of current environmental conditions (Lu et al., 2019). Thus, maternal and fetal stress system calibration are inherently intertwined, and the fetus is an active contributor (Del Giudice, 2012). Fetal signals to maternal stress physiology occur through multiple mechanisms, including fetoplacental hormones (e.g., placental CRH), fetal motor activity, and fetal microchimerism, the latter of which refers to the migration of fetal cells to maternal organs and tissues, including the maternal brain (for a review, see Glynn et al., 2018). The evolutionary-developmental maternal-fetal conflict theory posits that maternal and fetal interests are not always aligned: what is adaptive for the mother may not necessarily be adaptive for the fetus, and vice versa (see Fowden & Moore, 2012). While much of gestational biology involves maternal-fetal cooperation, the genetic interests of mother and fetus are only partially overlapping, and the level of resources the fetus demands may exceed what is optimal for the mother’s health and long-term reproductive strategy (Fowden & Moore, 2012; Haig, 1996). Maternal-fetal conflict theory proposes that each member of the dyad (unconsciously and at the level of biology) attempts to maximize its own fitness, with mother and fetus using physiological signals to manipulate resource allocation and the trajectory of gestation (Del Giudice, 2012; Gluckman et al., 2019). The maternal stress response is proposed to be a mechanism of maternal-fetal conflict, where its activity is modulated to influence the cues the fetus receives about the quality of the environment, as well as the degree to which the fetus is responsive to postnatal maternal inputs, to the benefit of the mother (see Hartman & Belsky, 2018; Lu et al., 2019). Interestingly, several studies link maternal ELS with trajectories of placental CRH over gestation (e.g., Steine et al., 2020), reflecting a possible mechanism of the intergenerational transmission of early adversity. In line with the notion that environmental information encoded by the SRS feeds back on the long-term calibration of the system over development (Del Giudice et al., 2011), perinatal maternal stress recalibration is likely to be constrained to a degree by the effects of environments during previous periods of calibration, with the fetus then receiving information about current and historical maternal life conditions. Ultimately, maternal-fetal conflict theory would argue that the fetus substantively participates in calibrating the maternal stress system, suggesting that perinatal recalibration must ultimately be examined from an intergenerational, dyadic perspective.

Finally, while stress (re)calibration theory and research focuses specifically on HPA axis responsivity, it is possible that the other components of the stress system (i.e., basal activity of the HPA axis, function of SAM axis), as well as other environmentally sensitive physiological systems, are adaptatively calibrated during developmental switch points, potentially in coordination. Ellis et al. (2021) recently expanded the ACM to propose that the oxytocinarginine vasopressin (OT-AVP) system is an additional mechanism of adaptive calibration of LH-related physiology and behavior. The OT-AVP system is critically involved in social bonding, attachment, and parenting behavior (Galbally et al., 2011). Meta-analytic evidence indicates that this system is responsive to environmental conditions during early life, with ELS relating to lower levels of endogenous OT (Ellis et al., 2021). In pregnancy and lactation, structural and functional plasticity is observed in OT neurons which project between brain regions subserving caregiving behavior (Kim & Strathearn, 2016), and oxytocin levels are linked with measures of attachment and caregiving behavior (Galbally et al., 2011). OT has inhibitory effects on the stress response (Heinrichs et al., 2003) and appears to be involved in the lactation-induced blunting of HPA axis responsivity in both rodents and humans (see Brunton et al., 2008; Slattery & Neumann, 2008), highlighting its possible mechanistic involvement in stress recalibration. Given the apparent plasticity of the OT-AVP system during pregnancy and lactation, this period plausibly represents a candidate window for recalibration of this system, which could serve as a mechanism for repair of social relationships following ELS (see Doyle & Cicchetti, 2017). The immune system is another candidate, as it is shown to be influenced by early life and perinatal environmental conditions (Aschbacher et al., 2021), is significantly altered in pregnancy (Abu-Raya et al., 2020), and appears to contribute to perinatal brain plasticity (Pawluski et al., 2022).

### Possible strategies for testing the hypothesis

Investigating the impacts of early life and perinatal environments in humans is inherently difficult, as random assignment to varying degrees of environmental stress and support is not possible, nor is there a high likelihood of significantly changed conditions from early life to adulthood. This creates the potential for many confounding factors. Possibilities for empirical investigation into the perinatal stress recalibration are now outlined, including non-human animal models, quasi-experimental human studies, and observational human studies.

### Non-human animal models

Animal-human translational studies will play a fundamental role in testing for perinatal stress recalibration. Animal studies afford multiple empirical benefits, though, direct translation of their findings to human studies is inherently limited by cross-species differences (including, as stated, that the rodent placenta does not produce CRH; Power & Schulkin, 2006). A rich history of experimental animal research has generated causal evidence of the consequences of ELS for SRS function and the mechanisms by which adversities (e.g., deprivation, abuse) become biologically embedded (see Lupien et al., 2009), offering “proof of principle” that causal effects may be at play in humans (Miller et al., 2011). Findings from ELS studies are remarkably similar across rodents, monkeys, and humans (see Callaghan et al., 2014), likely in part due to the highly conserved nature of the neuroendocrine SRS across species (Crespi & Denver, 2005). Non-human animal models of perinatal stress recalibration will allow for experimental manipulation of the quality of environmental conditions during early life and the perinatal period in a time-limited fashion, so that the opening and closing of sensitive windows of plasticity (Takesian & Hensch, 2013), the impacts of changed conditions (e.g., Morley-Fletcher et al., 2003), and the degree of change required for recalibration of the stress response can be elucidated. However, it will be important to bear in mind that such environmental manipulation would likely translate to fairly extreme conditions in humans.

Existing research in rodents illustrates how gestational stress alters adaptive neural plasticity during pregnancy and lactation (Slattery & Hillerer, 2016), which, taken together with mechanisms shown to underlie normative SRS alterations in pregnancy, reveals some of the neural pathways by which perinatal stress recalibration could occur. For example, repeated restraint stress in pregnancy increases hippocampal cell proliferation (opposite the typical pattern of decrease; Pawluski et al., 2015), an effect that appears to diminish shortly after weaning (Pawluski et al., 2012). Stress in pregnancy also decreases GR density in the CA3 region of the hippocampus, contrary to the increases in GR mRNA in the dentate gyrus characteristic of normative pregnancy (Johnstone et al., 2000; Pawluski et al., 2015). Further, postpartum stress interferes with the typical lactation-induced decrease in hippocampal volume and cell proliferation (Hillerer et al., 2014). Critically, these effects are not seen in nulliparous females, providing evidence that brain plasticity is differentially impacted by stress during pregnancy and lactation. Additionally, stress during the perinatal period has long-term consequences for neural processes such as hippocampal function and memory, while the impacts of these same stressors on non-perinatal rodents do not appear to persist with time (consistent with the notion that the impacts of stress experienced outside of sensitive periods can remit; Lemaire et al., 2006; Leuner et al., 2014). Stress-induced elevations in corticosterone are shown to contribute to these differences (Brummelte & Galea, 2010; Workman et al., 2013). These findings suggest that stress during the heightened neural plasticity of pregnancy and lactation may have stronger and more lasting effects, providing some indirect evidence in support of the pregnancy recalibration hypothesis.

Fewer rodent studies have investigated whether enhanced environmental supports during the perinatal period shape maternal brain plasticity and behavior, over and above the enrichment and cognitive challenges that early parenting inherently offers (see Orchard et al., 2023; Pawluski et al., 2016). Sparling et al. (2010) describe a perinatal enrichment paradigm for mother rats called a “social colony,” consisting of a network of cages that offers various physical enrichments as well as socially enriching interactions with other dams and their litters. This environmental enrichment predicts maternal behaviors, including indicators of faster acclimatization to novel situations. Such a paradigm could be used to examine the effects of supportive perinatal environments on long-term patterns of maternal HPA axis responsivity, including among rats raised in either stressful or supportive early life conditions.

In terms of the impacts of early life experience on perinatal plasticity, postpartum rats reared in social isolation and those raised by their mothers show comparably higher brain weights and altered dendritic complexity in several brain regions, suggesting that reproductive experience offers brain plasticity regardless of early life social conditions (Shams et al., 2012). Related research finds that pregnancy involves an increase in cortical depth that is *more pronounced* among rats raised in impoverished environments relative to those reared in enriched environments (Diamond et al., 1971; Hamilton et al., 1977). Other evidence suggests that maternal brain systems may be differentially organized in ELS-exposed versus non-exposed rats (Akbari et al., 2007; Shams et al., 2012). Collectively, these studies suggest that perinatal neural plasticity mechanisms may differ as a function of early life experiences.

Optimally, cross-species approaches can align mechanistic rodent studies with observational human data to substantiate the possibility for perinatal stress recalibration in humans. An excellent and relevant translational study considers how stress during preadolescence interacts with the dynamic hormonal milieu of pregnancy (conceptualized as a “second hit”) to alter stress responsivity in both mice and humans (Morrison et al., 2017). Female mice exposed to chronic variable stress during preadolescence show decreased cortisol responsivity to restraint stress during pregnancy, whereas preadolescent stress has no impact on cortisol reactivity in nulliparous female mice. In parallel, at 6 months postpartum, human mothers reporting a higher number of adverse childhood experiences show an attenuated cortisol response to an infant separation stressor relative to women with no reported adverse childhood experiences. Analyses of gene expression in the pregnant female mice implicate central rather than peripheral influences of preadolescent stress on gestational HPA axis function, including involvement of allopregnanolone (Morrison et al., 2017, 2020). Similar translational models would be very useful to test for perinatal stress recalibration and can allow for stronger conclusions to be drawn about non-experimental findings in humans.

### Human studies

Longitudinal assessment spanning pre-pregnancy to several years after the perinatal window would provide the within-individual data needed to assess whether women whose environmental conditions have shifted significantly from earlier periods of calibration show changes in stress responsivity from pre- to post-perinatal period that persist for several years. Such a cohort could be paired with a nulliparous control group followed at matched intervals, with the hypothesis that the control group would not demonstrate changes in stress responsivity to the same extent or to any meaningful degree, even if they have experienced significant shifts in environmental conditions from previously. Such a study would be a massive and logistically difficult undertaking. One possibility would be to leverage existing prospective, longitudinal cohort studies which have characterized environmental conditions and HPA axis activity beginning in childhood and into reproductive age. More robust adjustment for confounds using propensity score modeling (see Martín-de-las-Heras et al., 2022) or co-twin control (see Turner et al., 2020) methods could strengthen causal inference. As a necessary first step, components of the perinatal stress recalibration hypothesis can be tested. For example, a co-twin control study could compare profiles of HPA axis responsivity among twin pairs raised in the same early environment, where one twin experiences a perinatal period and the other has no history of such period (with potentially discordant current environmental conditions).

Another alternative in humans is to use the “intervention as a test of mechanism” approach (see Cicchetti & Gunnar, 2008), a strategy particularly suitable for examining whether stress recalibration occurs if conditions shift from harsh to supportive. Individuals cannot be randomly assigned to experience stress, but random assignment to intervention is feasible. Pregnancy is already recognized as an opportune time for intervention, as it offers heightened neural plasticity (Davis & Narayan, 2020; Glynn et al., 2018), involves greater engagement with the healthcare system, is associated with spontaneous positive behavior change (Massey & Wisner, 2018). Establishing that interventions which improve environmental conditions have a larger impact on the SRS during pregnancy and/or lactation as compared to outside of the perinatal period would speak to the neural plasticity of pregnancy and the heightened sensitivity of the stress system to environmental conditions during this time. In the broader developmental literature, neurobiological measures are integrated into intervention research to document that interventions can reverse or prevent HPA axis dysfunction (Cicchetti & Gunnar, 2008). For example, children living in foster care randomly assigned to therapeutic interventions exhibit cortisol profiles that become comparable to those of non-foster care children over the intervention, whereas foster care children receiving care as usual continue to show distinct cortisol patterns (e.g., Dozier et al., 2008). Relevant to perinatal stress recalibration, women at high risk for depression assigned to receive intervention show more typical diurnal cortisol patterns from pregnancy until 18 months postpartum and lower cortisol levels at 18 months postpartum (Urizar & Muñoz, 2011). Critically, intervention science must bear in mind the issue of phenotype-environment mismatch. If maternal and fetal stress systems adaptively calibrate to match supportive conditions offered by the intervention, but conditions revert to unsupportive after the intervention concludes, risk for maladaptive outcomes is likely to increase. This potential issue highlights the need for interventions to be designed to ensure that supports are *sustained*.

## Conclusions

Adaptive calibration of the neuroendocrine SRS is a primary proposed mechanism by which social and physical environments experienced during early life sensitive windows of heightened neural plasticity shape physiology and behavior across development (Del Giudice et al., 2011). Emerging evidence in humans, bolstered by experimental non-human animal research, suggests that responsivity of the HPA axis is subject to recalibration during puberty, another window of heightened plasticity and normative shifts in SRS activity (see DePasquale et al., 2021). The perinatal period is yet another life phase characterized by enhanced neural plasticity and substantive alterations in HPA axis basal activity and responsivity, and a growing literature suggests that HPA axis functioning during this period reflects both early and current life environmental conditions. The perinatal period may therefore constitute an additional window of stress system recalibration during which lifespan and intergenerational developmental trajectories can be redirected, with recalibration plausibly serving as a multilevel, multisystem mechanism risk and resilience. Testing the pregnancy recalibration hypothesis will be a complex venture for a myriad of reasons highlighted above, and multidisciplinary, cross-species approaches will be key in beginning to understand this possibility.

The perinatal stress recalibration hypothesis is aligned with a life-course perspective (Lu & Halfon, 2003) and with broader calls to conceptualize pregnancy as a sensitive window of both heightened vulnerability and opportunity for positive change (Davis & Narayan, 2020; Glynn et al., 2018; Howland & Cicchetti, 2021). If the perinatal period opens a window for recalibration of a key body system that regulates physiology and behavior at multiple levels, both intra-individually in the lifespan of the birthing parent and intergenerationally for the developing fetus/infant, this suggests that this life phase should be leveraged to optimize the impacts of resilience-promoting preventions and interventions. Many birthing individuals who experience environments characterized by harshness (threats of violence, material deprivation) and unpredictability (housing instability, tenuous family relationships) in early life and/or in reproductive age face systemic barriers (e.g., poverty, racism, historical trauma) which limit opportunities to foster supportive conditions for healthy development. Therefore, in addition to identifying individual-level perinatal protective and promotive factors to inform interventions (Davis & Narayan, 2020), it will be critical to address the multiple broader systems and social inequities which foster adverse environmental conditions (Howell et al., 2021; Lu & Halfon, 2003). Attending to these macrolevel factors is likely to yield more sustainable intervention effects, which can improve the lifespan health of women and disrupt intergenerational cycles of adversity.
